# Abstracts From the Annual Meeting of the International Infectious Disease Society for Obstetrics and Gynecology USA

**DOI:** 10.1155/S1064744997000124

**Published:** 1997


Infectious Diseases in Obstetrics and Gynecology 5:57–67 (1997)
© 1997 Wiley-Liss, Inc.

Abstracts From
the

### Annual Meeting of the International Infectious Disease Society for Obstetrics and Gynecology USA

Las Vegas, Nevada

April 25–26, 1997

Special Section

### FRIDAY, APRIL 25TH, 1997

### First Scientific Session

1:00

### PREVALENCE OF NON-ALBICANS SPECIES IN ACUTE VULVOVAGINAL CANDIDIASIS

J. Gunter*, K. Ault, S. Faro, Department of Obstetrics and Gynecology, Universityof Kansas Medical Center, Kansas City, Kansas.

Objective: Determine the prevalence of non-albicans species in acute vulvovaginal candidiasis.
Study Design: A prospective studyof 82 women with acute vulvovaginal candidiasis and 76 matched controls was performed. Women presenting with appropriate symptoms (abnormal vaginal discharge, pruritis, or burning) and either vulvar erythema or abnormal vaginal discharge identified on physical exam were evaluated via vaginal pH determination and smears for saline and potassium hydroxide microscopy. A diagnosis of acute vulvovaginal candidiasis was made in those women with a pH <4.4, the presence of buds, mycelia or pseudohyphae on microscopy. Cultures were collected from the lateral side walls and posterior fornix on Saborauds dextrose slants and identified by standard methods. Seventy-six consecutive controls from a contraception clinic had cultures performed as described. The patient and control populations were equally matched for age, parity and method of contraception.
Results: Fungal cultures were positive in 61/82 (72.4%) women with clinically diagnosed acute vulvovaginal candidiasis. A total of 70.5% of the isolates were *C. albicans*; 18% *C. glabrata*; 3.3% *C. tropicalis*; 3.3% *C. krusei*; 3.3% *C. paropsilosis*; 1.6% *S. cerebaciae*. Positive fungal cultures were identified in 15 of the 76 asymptomatic controls (19.4%), 67% were *C. albicans* and 33% were *C. glabrata*.
Conclusions: Non-albicans strains while responsible for a significant number of acute vaginal fungal infections, were isolated with the same frequency from asymptomatic women with positive cultures. Fungal cultures, whil an important adjuvant tool, must be interpreted with caution as only 72.4% of women with clinically diagnosed acute vulvovaginal candidiasis have positive fungal cultures.

1:20

### VAGINAL ALLERGY, IMMUNE SUPPRESSION AND SUSCEPTIBILITY TO RECURRENT CANDIDA VAGINITIS

Steven S. Witkin Cornell University Medical College, New York, New York

The immune response to Candida albicans is influenced by genetic factors and the concurrent presence of immune activators or inhibitors. Many women with recurrent Candida vaginitis had poor *in vitro* lymphocyte proliferative responses to this organism. Inclusion of inhibitors of prostaglandin (PG) synthesis restored normal responses indicating that induction of high levels of PG inhibited immunity in these women. The women with recurrent Candida vaginitis also had a high prevalence of anti-Candida IgE in vaginal secretions and eosinophils in vaginal smears. Other symptomatic women had IgE to semen, contraceptive gels, or environmental allergens. An allergic response triggered histamine release which resulted in the stimulation of PG production by macrophages. Furthermore, histamine and C. albicans were shown to be synergistic for enhanced PG induction from macrophages. Thus, if C. albicans happened to be present in the vagina at the time of an allergic response the combination of Candida plus histamine resulted in very high levels of PG release and a suppression of local immunity. The C. albicans then readily proliferated and induced clinical symptoms. Treatment of only the C. albicans infection would not reduce susceptibility to allergy-mediated immune suppression and so these women would remain at high risk for repeated episodes of Candida vaginitis.

1:40

SIGNIFICANCE OF WHITE BLOOD CELLS IN ASSOCIATION WITH PSEUDOMYCELIA IN WET MOUNT PREPARATIONS

Gilles RG Monif, MD, Omaha, Nebraska

Objective: The purpose of this paper if to analyze the significance of concomitant presence of inflammation with the tissue invasive form of Candida albicans.

Material and Methods: All wet mount/potassium hydroxide (KOH) forms from January 1994 through December 1995 were reviewed for the presence of pseudomycelia on one or both preparations. Those reports containing definite pseudomycelia were reviewed specifically for 1) the quantity of WBC's concomitantly present, 2) the presence or absence of clue cells , 3) the presence or absence of lactobacillus and 4) the presence or absence of a positive volatile amine test.

Results: Within the 1,471 wet mounts/KOH preparation derived from women seen at the Women's Clinic and St. Joseph Hospital, 207 cases were identified in which pseudomycelia were present. Reports in which only blastospores or budding elements were identified were not included in the criteria. Out of the 207 cases in which pseudomycelia were present, 159 had quantitation of the number of white blood cells per high power field concomitantly present. One hundred and two (64%) of patients had no significant inflammation (63 had less than 2 WBC/hpf, 39 had less than 3-5 WBC/hpf). Thirty-five patients (22%) had minimal to moderate inflammation (5-10 WBC/hpf). Twenty-two (13.8%) had greater than 10 WBC/hpf. Seventeen patients had either clue cells or positive volatile amine tests. The majority of patients with significant inflammation and another disease process present.

Conclusion: Presence of inflammation associated with the tissue-invasive necessitates the quest for additional disease states which, if present requires individualization of therapy.

2:00

LONGITUDINAL INVESTIGATION OF CANDIDA VAGINITIS SYMPTOMS IN PREGNANCY: ROLE IF SUPERIMPOSED ANTIBIOTIC USE.

Douglas D. Glover and Bryan Larsen. West Virginia University, Morgantown, WV and Marshall University, Huntington, WV.

A commonly recited tenet of medicine is that antibiotic use is often complicated by vulvovaginal candidiasis (vvc). We wondered how strong this association is and whether it is applicable to current prescribing practices. We undertook a prospective, longitudinal culture-based observational study of 250 women followed from their first prenatal visit through their six weeks postpartum visit, documenting antibiotic use, complaints, signs and symptoms of yeast vaginits. Vaginal culture for Candida was done at the first visit and in each patient who developed symptoms. Patients symptomatic at their first visit were excluded from this evaluation. Study participants were categorized as colonized or uncolonized based on their first visit culture. We found a significantly increased risk of developing symptoms among women who were asymptomatically colonized. For the entire period of observation there was a 10.9% risk of any patient becoming symptomatic, but a 5.1% risk of becoming symptomatic after antibiotic treatment. Among the patients in our study, 43% were treated with at least one course of antibiotic and several patients were treated with multiple courses of antibiotics without developing vaginitis symptoms. Antibiotics were prescribed by family practicioners, emergency room physicians, dentists, dermatologists or surgeons, illustrating the need for obstetrician to be aware that their obstetrical patients may use various drugs without the advice of the obstetrician. All cases of symptomatic infection occurred prior to delivery and no cases were seen between delivery and the 6 week postpartum visit, indication that pregnancy and vaginal colonization are more significant risk factors for symptomatic vvc, than is antibiotic treatment.

2:40

RECOLONIZATION WITH LACTOBACILLI IN PATIENTS WITH CHRONIC VAGINAL INFECTIONS WITH THE USE OF INNER CONFIDENCE™... A FEMALE CONTROLLED VAGINAL MICROBICIDE

Larry C. Ford[1,2], Valerie P. Kesler[2], Hunter A. Hammill[3], and Thomas e. Lebberz[4], OB-GYN, UCI, Orange, CA[1], Lafor Laboratories, Newport Beach, CA[2], OB-GYN, Baylor Houston, TX[3], OB-GYN, UCLA, Los Angeles, CA[4]

Previously we have reported our observations that inpatients with chronic vaginitis (longer than three months duration), and most frequently, irrespective of the etiology(ies) of the initial vaginal infection, if known, most frequently have low or nondetectable levels of lactobacilli and high levels of enterics and anaerobic organisms. These patients are often difficult to successfully manage clinically. Even though the importance of the lactobacilli has been known for over one hundred years, it is difficult to re-establish it as the dominant flora in such patients. There are and have been numerous preparations of lactobacilli which have been touted to cause the recolonization of these beneficial bacterial in the lower genital tract. However, the success of such simple products, for numerous reasons, have not been clinically useful in most patients.

It is possible that these simple lactobacilli products fail to "recolonize" because of changes in the vaginal pH, alterations in cellular adherence, altered metabolic or host defenses interactions, etc. We postulate on the basis of quantitative culture that the already well established and more rapidly reproducing microorganisms such as E. coli, simply overgrow the lactobacilli introduced in any vehicle or carrier. We further postulate that it is essential to markedly reduce the levels of the enterics and other related organisms by the use of safe, topical antimicrobials so that the appropriate lactobacilli can be successfully reestablished as the dominate flora.

The polyantimicrobials in Inner Confidence™ eradicate or at least lower the numbers of these "opportunistic" organisms which is requisite to the reestablishment of the lactobacilli, which can produce hydrogen peroxide and microcidins. We have found that the use of Inner Confidence™ re-establishes such healthy lactobacilli as the dominant flora.

3:00

INHIBITORY EFFECT OF COMMERCIAL DOUCHE PRODUCTS AGAINST VAGINAL MICROFLORA.

Sylvia I. Pavlova*, Susan M. Mou and Lin Tao. University of Missouri-Kansas City, Kansas City, MO64108

OBJECTIVE; Although the cause of bacterial vaginosis (BV).is unknown, during BV a reduction in vaginal lactobacilli occurs. Recently, BV has been associated with vaginal douching, suggesting that douching may affect the concentration of lactobacilli in the vagina. The aim of this study was to analyze the antimicrobial effect of commercial douche products on various vaginal bacteria, including lactobacilli.

STUDY DESIGN: Seven different commercial douche products were included in the study. Eight vaginal *Lactobacillus* strains isolated from asymptomatic women and 3 vaginal *Lactobacillus* strains from the American type Culture Collection representing 6 different *Lactobacillus* species were tested *in vitro* for their sensitivity to these douche products. Strains representing six vaginal bacterial genera, *Gardnerella, Mobiluncus, Mycoplasma,* group B *Streptococcus, Peptostreptococcus* and *Ureaplasma,* presumably associated with BV were also tested. The minimal inhibition concentrations and minimal contacting times for these products to inhibit bacteria were determined in broth cultures.

RESULTS: Four of the 7 douche products showed a strong antibacterial effect against vaginal lactobacilli with a very short contact time (less than 1 min). The six suspected BV pathogens tested were more sensitive to these products than vaginal lactobacilli. However, 2 douche products made of water and vinegar did not affect the growth of vaginal microflora. One douche product had only a moderate antibacterial effect.

CONCLUSION: The use of douche products that contain antibacterial agents could alter the composition of vaginal microbial flora, including lactobacilli.

3:20

### ONCE DAILY MODIFIED-RELEASE ORAL METRONIDAZOLE IS MORE EFFECTIVE THAN CLINDAMYCIN VAGINAL CREAM IN THE TREATMENT OF BACTERIAL VAGINOSIS

David A. Baker, MD, James A. McGregor, MD, CM, and Gilles R.G. Monif, MD, Health Science Center, State University of New York, Stony Brook, NY; University of Colorado Health Sciences Center, Denver, CO; Creighton University, Omaha, NE.

A modified-release formulation of metronidazole (Flagyl MR, Searle MR) has been developed to permit once-daily dosing. Two multicenter, randomized, investigator-blind studies compared MR and 2% clindamycin vaginal cream (CVC) in women with bacterial vaginosis. This report represents pooled data. A total of 709 patients (ages 16 to 68 years) were randomized to receive 750 mg MR orally QD for 5 days (n=152) or 7 days (n=270), or 1 g CVC QD for 7 days (n=287). Outcomes were assess 4 to 7 days (Visit 2) and 28-32 days (Visit 3) after treatment. Clinical cure was defined as resolution of Amsel's criteria. At Visit 2, dichotomized (cure/not cure) clinical cure rates were 58% for intent-to-test patients in the MR 5-day arm, 59% in the MR 7-day arm ($p<0.0001$ for both MR arms vs. CVC), and 32% in the CVC arm. Normal vaginal flora (Spiegal criteria) was noted in 76% of patients in the MR 5-day arm, 81% in the MR 7-day arm, and 51% in the CVC arm ($p<0.0001$ for both MR arms vs. CVC). At Visit 3, clinical cure was noted in 51% of patients in the MR 5-day arm ($p>0.05$ vs. CVC), 56% in the MR 7-day arm ($p=0.023$ vs. CVC), and 47% in the CVC arm. Normal vaginal flora was noted in 67% in the MR 5-day arm ($p>0.05$ vs. CVC), 74% in the MR 7-day arm ($p=0.0049$ vs CVC), and 63% in the CVC arm. MR and CVC were well tolerated. There were no statistically significant differences in the incidence of yeast vaginitis.

In summary, MR orally QD for 5 or 7 days is an effective treatment of bacterial vaginosis, providing significantly more rapid clinical cure, normalization of pH, and return to normal, Lactobacillus-dominant vaginal flora than 2% clindamycin vaginal cream.

### SATURDAY, APRIL 26, 1997

### Second Scientific Session

8:00
THE VALIDITY OF MULTIPLE SITE SAMPLING FOR THE DETECTION OF GROUP B STREPTOCOCCI IN PREGNANT WOMEN
Michael S. Burnhill, M.D., D.M. Sc.*

Introduction: To prevent neonatal disease, pregnant women are screened for Group B streptococci; the specimen for GBS cultures is routinely obtained from the vagina. However, while the superiority of lower versus upper vaginal cultures has been documented, few studies have examined the validity of a single culture site This study compares different sampling sites in women positive for GBS
Methods: Simultaneous cultures were obtained from the rectum, mid-perineum, vagina. and urethra, and comparisons between sampling sites were made for women positive in at least one culture for GBS.
Results: The outcome of sampling the urethra, mid-perineum, rectum and vagina will be presented for each site and multiple site comparisons will be made.
Conclusion: In the future, multiple-site sampling can detect more women colonized with GBS than single site sampling of the vagina, thereby reducing the rate of false-negative tests for Group B streptococci.

8:20
EARLY GESTATIONAL BLEEDING AND BACTERIAL VAGINOSIS INCREASE RISKS OF PRETERM BIRTH;
Janice I. French, James A. McGregor* MD CM, Deborah Draper, Ruth Parker, John McFee MD, University of Colorado School of Medicine, Denver, CO USA

Evaluate associations between prevalent lower genital infections (LGTI) and first trimester bleeding on birth outcomes. 1,260 inner city women were evaluated in a controlled prospective trial of standardized diagnosis, treatment and follow-up for women with LGTI. Stratified analysis was performed First trimester bleeding (11%), bacterial vaginosis (BV) (3 1 %) and trichomoniasis (TV) (9.5%) were common. Among women with BV alone (n=214), bleeding (BLD) was associated with increased PTB (28%) compared to women without bleeding (9.0%) (RR=3. 1, CI 1.4-6.8). In the absence of BV, BLD was associated an insignificant increase in PTB (BLD=13.5% vs. No BLD:8.4%; p=.3; RR=1.6, CI 0.7-3.8). Women with both BV and TV (n-31) suffered increased PTB in the presence of bleeding (BLD:44.4% vs. No BLD:22.7%; RR-2.0, CI 0.7-5.6). BV treatment was associated with improved birth outcome primarily ill women who had not reported first trimester bleeding. Fewer women who had not reported first trimester bleeding. Fewer women had TV alone; treatment of TV was also associated with reduced PTB in women without bleeding. Early gestational bleeding was associated with PTB in the presence of BV alone or BV and TV. Uterine bleeding may be: (1) caused by genital infection; (2) a permissive factor amplifying effects of infection; or (3) an independent risk factor for PTB. Women with early bleeding should be screened and treated for BV and TV. The ideal time to identify and treat BV and TV may be prior to conception.

8:40

### PREGNANCY, UROGENITAL INFECTION AND MICROBIAL INVASION

Pawel Goluszko, Tuan Pham, Rangaraj Selvarangan, Iyotsana Singhal, Stella Nowicki, Bogdan J Nowicki*. The University of Texas Medical Branch. Galveston, Texas 77555-1062.

The underlying cellular and molecular mechanisms of urogenital infections in pregnant women are not well understood. Colonization of the lower genital tract is considered as an important step in ascending infection. We have recently shown *E. coli* that express colonization factor Dr fimbriae are predominant in pyelonephritis during the last trimester. Following this report, we found that uterine infection of pregnant animals with Dr+ *E. coli* resulted in lethal outcome. Dr+ *E. coli* disseminated to kidneys and other organs. In this study, we evaluated the possible invasive properties of Dr+ *E. coli* observed in pregnant rats. A cervical carcinoma cell line, HeLa (which express Dr receptor) was used to test virulence of Dr+ *E. coli* in a standard invasion assay and transmission electron microscopy. Dr+ *E. coli*, both clinical and recombinant that express Dr fimbriae were found to be internalized to HeLa cells at the rate of 4000 to 6000 CFU per well. Inhibition of invasion with anti-DAF or anti-Dr fimbriae IgG was observed following blocking of HeLa cell receptor DAF (75%) and Dr fimbriae ( 100%), respectively. Mutation of the *E. coli* structural fimbrial gene *draA* abolished invasion (100%~. We conclude that Dr+ *E. coli* which are predominant in the III-trimester pyelonephritis. possess capacity to invade epithelial cells of the lower genital tract. We consider that invasion of the epithelial cell of the lower genital tract may contribute to persistent colonization of the lower genital tract and may increase the risk of gestational infection.

9:00

### EVIDENCE BASED PREVENTION OF PRETERM BIRTH/PROM: INFECTION AND INFLAMMATION;

James A. McGregor* MD CM, Janice I. French, University of Colorado School of Medicine, Denver, CO USA

Prevention of preterm birth and subsequent newborn immaturity continues as a primary goal of health care. Considerable information shoes that (1) 25%-50% of preterm births are caused by common genital tract infections and subsequent maternal/fetal inflammatory responses; (2) both microbial and host factors (phospholipases, proteases, etc.) play complex and synergistic roles in preterm labor and premature rupture of membranes (PROM); (3) expedited identification and systemic treatment of common genitourinary infections, most importantly bacterial vaginosis (BV), can consistently reduce risks of preterm delivery and PROM; and. (4) antimicrobial treatment with erythromycin, clindamycin or other antibiotics can significantly delay delivery and reduce risks of maternal and neonatal morbidity in women with preterm labor or PROM. Diagnosis of BV can be made using clinical criteria; homogeneous discharge; pH>4_7. positive urine test, and "clue" cell identification (3 of 4 criteria). Optimal treatment for BV during pregnancy is either oral clindamycin or metronidazole followed by "test of cure" evaluation and retreatment necessary. Both asymptomatic and symptomatic women should be treated. Interactions of reproductive tract infection and inflammation with other obstetric factors including short cervix, multiple gestation, and maternal smoking will be discussed. Medical care providers now have the opportunity as well as the obligation to prevent infection-mediated preterm birth and to optimize the care of each mother and baby.

9:20

### EXPRESSION OF TISSUE RECEPTORS FOR E. COLI VIRULENCE FACTOR (P-FIMBRIAE) IN THE C3H/HEJ MICE KIDNEY DURING PREGNANCY.

Anil Kaul M.D.[1,2], V. Lupo M.D.[2], Shah Khan Ph.D[2], Rashmi Kaul Ph.D1,[2], Bharat Kachroo Ph.D.[1], Mark Martens M.D.[12], Ob/Gyn Department [1]Univ of Minnesota, and [2]Minneapolis Medical Research Foundation, Minneapolis, MN.

Objective: There is a significant increase in the incidence of pyelonephritis in pregnant population. The pathogenesis of pyelonephritis in non-pregnant population has been associated with bacterial virulence factors, such as 'P' fimbriae, present in about 70-90% of all pyelonephritis cases. We hypothesize that during pregnancy there is an increase in the expression of host receptors for 'P' fimbriae of uropathogenic *E. coli* and this increase may result in an increased colonization leading to an increased incidence of pyelonephritis during pregnancy.
Study Design: Kidney sections obtained from C3H/HeJ mice at difference gestational ages were fixed in acetone and incubated with FITC labelled *E. coli* strains expressing pap-1 and pap-2 fimbriae for one-hour and after washing with PBS, sections were viewed under fluorescent microscope.
Results: Significant increase in the attachment of pap-1 fimbriated E. coli was observed during the first half of pregnancy whereas there was increased attachment of pap-2 during the second half of the pregnancy.
Conclusions: The dynamic change in the attachment of pap1 and pap2 fimbriated E.coli to C3H/HeJ mice kidney appears to be in agreement with our hypothesis that there may be an increase in the expression of specific tissue receptors during pregnancy that promote colonization by E. coli bearing these specific virulence factors. We, therefore, propose that over-expression of tissue receptors may be an important factor in the pathogenesis of pyelonephritis in pregnant population.

10:00

### AMPICILLIN RESISTANCE IN DR FIMBRIAE BEARING UROPATHOGENIC *ESCHERICHIA COLI*

BB Kachroo Ph.D[2], Rashmi Kaul Ph.D[1,2], Shah Khan PhD[2], D.Saryavolu M.D., Mohammad El-Zataari M.D[1]., Mark Martens M.D.[1,2], Anil Kaul M.D.[1,2], Ob/Gyn Department [1]Univ of Minnesota, and [2]Minneapolis Medical Research Foundation, Minneapolis, MN.

Microorganisms like *Escherichia coli* express lectin-like surface structures called fimbriae, pili, or adhesins that mediate attachment of pathogens to various tissues through specific receptors/ligands. Adherence to the tissue substructures facilitated by adhesins is considered to be a prerequisite step for initiation of the disease process and infection of the host. One such adhesin called Dr is present on uropathogenic *E. coli*.
In the present study, we investigated if there was any correlation between the expression of fimbriae and other virulence factors like antibiotic resistance among uropathogenic *E. coli*.
A total of 161 E. coli isolates were collected and typed for fimbrial expression by hemagglutination. All the isolates were tested for ampicillin resistance and their percentage is given in the following table(n)

| Groups/Sub-Groups | Study I | Study II |
|---|---|---|
| Total Isolates | 38.55% (83) | 38.46% (78) |
| 1) Non-HA | 32.43% (37) | 27.27% (44) |
| 2) MSHA | 43.47% (46) | 52.8% (34) |
| a) Type 1 | 50% (10) | 20% (5) |
| b) MRHA | 41.66% (36) | 48.27% (29) |
| **i) Dr** | **72.72% (11)** | **75% (8)** |
| ii) Non-Dr | 28% (25) | 38.09% (21) |

Nearly 75% of all the Dr bearing uropathogenic *E. coli* were resistant to Ampicillin. Further studies are in progress to investigate if the presence of Dr fimbriae is the cause of the effect of Ampicillin resistance.

10:20

THE STORY OF TRICH IN PREGNANCY, AS TOLD BY THE VIP STUDY. Joseph C. Pastorek MD, Marcy Frances Cotch, Ph.D. From the Louisiana State University Department of Obstetrics and Gynecology in New Orleans and the National Institute of Allergy and Infectious Disease in Bethesda.

*Trichomonas vaginalis* has been considered a possible etiologic agent of adverse pregnancy outcome. The various demographic, behavioral, microbiologic and physical findings associated with trichomoniasis are not as well characterized as they are in nonpregnant women. The Vaginal Infections and Prematurity Study Group examined various factors in women colonized by T. vaginalis in mid pregnancy and determined the following: Women with trichomoniasis during pregnancy are more likely to be black, cigarette smokers, unmarried and poorly educated; T. *vaginalis* colonization was associated with increased lifetime sexual partners; trichomoniasis was associated with abnormal cervical/vaginal discharge and abnormal odor after addition of KOH; microorganisms associated with *T. vaginalis* include *Candida* spp, *Mycoplasma hominis, Chlamydia trachomatis, Ureaplasma urealyticum, Neisseria gonorrhoeae,* group B streptococcus, and *Bacteroides* spp; trichomoniasis in mid gestation was significantly associated with low birth weight and preterm delivery, especially in black patients. These data suggest that physical findings are not clinically helpful in the diagnosis of trichomoniasis in pregnancy other organisms need to be considered in any study of trichomoniasis, and treatment may have some (small) potential effect at preventing adverse pregnancy outcome due to trichomoniasis.

10:40

ULTRASOUND MARKERS OF INTRAUTERINE INFECTION

Newton G. Osborne, MD, PhD Howard University, Washington DC, USA; Luiz Antonio Bailão, MD, PhD Universidad de São Paulo, Facultod de Riberuo Preto, BRASIL; FernandoBonilla-Musoles, MD, PhD Universidad de Valencia, Valencia, Espana

Diagnosis of intrauterine infection has depended on positive cultures or immunologic techniques used to detect and identify microorganisms known or suspected to be associated with adverse pregnancy outcomes. Intrauterine infections with intact membranes can occur with viruses, bacteria, fungi, and protozoa. These infections may be overt with classical presentation in mothers, or they may be completely silent. In many instances, by the time infection in confirmed by culture results or by histopathological findings, fetal compromise may be so extensive the irreversible and permanent impairment or death in inevitable. Intrauterine infection can affect fetal development in subtle or obvious ways. Indicators of fetal infection may range from subtle fetal growth restriction, inappropriate development or calcification of fetal organs or of fetal adnexal structures (placenta and membranes), failure to develop fat stores or depletion of fat stores, to more obvious effects such as gross malformations or intrauterine fetal demise. Ultrasound is able to detect some of the subtle and most of the gross changes typical of fetal infections.

### Third Scientific Session

1:00

### SELF-INTROITAL SAMPLING FOR DETECTION OF *CHLAMYDIA TRACHOMATIS*

Jan Jeremias, Margaret Polaneczky, Vera Tolbert and Steven Witkin Cornell University Medical Center, New York, New York

Most women with occluded fallopian tubes never had symptoms of a chlamydial infection but have antibodies to *C. trachomatis*. Detection of asymptomatic carrier of *C. trachomatis* is essential to reduce the incidence of negative sequela of a chlamydial infection as well as decrease its rate of transmission. Screening women for *C. trachomatis* involves a speculum examination and endocervical sampling. This may be unacceptable or unavailable to some women at risk for infection. We evaluated whether self-collection of introital samples was an alternative to obtain specimens for *C. trachomatis* detection. An illustrated brochure was developed which prescribed the procedure for self-collection of introital samples. Patients consisted of 62 women attending an outpatient clinic, some of whom had tested positive for *C. trachomatis* by DNA probe on their previous visit and were returning for treatment. After the self-sample was collected, the clinician collected a second introital sample followed by an endocervical sample. All samples were tested for *C. trachomatis* by Amplicor polymerase chain reaction (PCR). The self-collected introital sample was positive for *C. trachomatis* in 17 (27.4%) women. These same women but none of the others were also positive in the clinician-collected introital sample. All but one of these women, and none of the others, were also positive for *C. trachomatis* in the endocervix. With proper instruction self-collected introital sampling is a viable alternative to endocervical sampling for *C. trachomatis* infections in women. The availability of this option should greatly increase the numbers of women for whom *C. trachomatis* testing can now be performed.

1:20

### FACTORS AFFECTING PHAGE INDUCTION IN VAGINAL LACTOBACILLI

Lin Tao*, Sylvia I. Pavlova, and Susan M. Mou. University of Missour-Kansas City, MO 64108

Objective: Bacterial vaginosis (BV) is associated with an unexplained reduction of vaginal lactobacilli. Previously, we reported that phage induction can lyse vaginal lactobacilli. The aim of this study was to analyze whether phage induction in vaginal lactobacilli can be promoted by various factors *in vitro*.
Study Design: Lysogenic vaginal Lactobacillus cultures were treated with heat shock (temperatures higher than 37° C), varied pH conditions, tobacco chemicals [tar, nicotine, benzol(a)pyrone, and benzol(a)pyrone diol epoxide (BPDE)], alcohol, vaginal douche solutions, a contraceptive foam with nonoxynol-9, and various sublethal concentrations of antibiotics. Phage titers of the cultures were analyzed by agar drop assay. Dairy lysogenic strains and Mitomycin C were used as controls.
Results: Among all factors tested, the cigarette carcinogen BPDE showed the highest effect in promoting phage-induction in vaginal lactobacilli, about 8 to 50-fold increase compared with their spontaneous phage induction rates. Antibiotics and the spermicide foam showed a moderate effect, about 3 to 10-fold increase. Among various antibiotics tested, kanamycin and penicillin had no significant effect, while erythromycin and novobiocin showed a 7-fold increase in phage induction. Conversely, various douche solutions, alcohol, varied pH conditions and increased temperatures did not affect phage induction in lactobacilli.
Conclusion: Smoking, antibiotic therapy and use of vaginal spermicide (nonoxynol-9) may increase the risk of reducing vaginal lactobacilli via phage induction.

1:40

INTERVAL BETWEEN CERVICAL CONIZATION AND VAGINAL OR ABDOMINAL HYSTERECTOMY WITH PROPHYLAXIS AND IMPACT ON THE INCIDENCE OF POSTOPERATIVE PELVIC INFECTION.

DL Helmsell, MD, ER Johnson, MD, P Hemsell, B Nobles. UT Southwestern Medical Center at Dallas, Parkland Memorial Hospital, 5323 Harry Hines Blvd., Dallas, Texas.

Most data relating to the interval between cervical conization and hysterectomy, and increased postoperative pelvic infection risk were collected prior to routine antibiotic prophylaxis at hysterectomy. General recommendations were that if hysterectomy could not be performed within 3 days, one should wait 6 weeks to decrease postoperative infection. No comparative data for vaginal and abdominal approach for benign disease can be found. Of 539 women undergoing conization/hysterectomy with prophylaxis in Parkland Memorial Hospital, 48 (8.9%) developed major pelvic infection. Of 422 women undergoing vaginal hysterectomy, 28 (5.9%) developed infection compared with 20/117 (17.1 %) women undergoing abdominal hysterectomy (p=0.001). After vaginal hysterectomy, any internal from 0 (frozen cone) to 120 days did not affect the postoperative infection rate (p=0.59), whereas interval after abdominal hysterectomy was important (p=0.05); 20/90 (22.2%) women with an interval from 0-18 days became infected postoperatively compared with 0/27 women whose interval was 19-120 days (p=0.003). Current surgery scheduling is based on these data.

2:00

WHAT IS REQUIRED FOR PROTECTION AGAINST SEXUALLY TRANSMITTED DISEASES AND OTHER CAUSES OF VAGINITIS?

Larry C. Ford[1,2], Valerie P. Kesler[2], Hunter A. Hammill[3] and Thomas B. Lebherz[4]. OB-GYN, UCI, Orange, CA[1], Lafor Laboratories, Newport Beach, CA[2], OB-GYN, Baylor, Houston, TX[3]. OB-GYN, UCLA, Los Angeles, CA[4]

It has been accepted since the late 19th Century that lactobacilli are the normal flora in almost all women. The metabolic activities of the lactobacilli, which include the maintenance of an acidic pH, the production of antimicrobial substances, enhancement of host resistance, as well as other favorable actions, have been studied for several years. It is well known that if the appropriate lactobacilli are scant or absent, the risk of acquiring certain infections are increased. Regrettable, women are susceptible to exogenous pathogens., especially STD's, even though they have "normal" flora. The appropriate protection can be life saving in our current era of HIV.

As reported previously, the frequent use of Inner Confidence™ does not cause vaginal lesions associated with the use of Nonoxynol-9 products. Inner Confidence™ destroys both the white blood cells and spermatozoa that may act as carriers of infectious organisms. It also prevents the post coital rise in vaginal pH and is compatible with latex condoms. Intravaginally, Inner Confidence™ kills Gardnerella vaginalis, Neisseria gonorrhoeae, the pyogenic cocci, the enterics, Candida species, Trichomonas vaginalis, Chlamydia trachomatis, Herpes simplex I and II, HIV, and other pathogenic and potentially pathogenic organisms. In addition to its polyantimicrobial activities, Inner Confidence's™ most important safety profile for women is the replenishing of the normal vaginal flora when the micro-encapsulated lactobacilli are released, producing hydrogen peroxide and microcidins.

